# Impact of Lower Airway Inflammation on Fitness Parameters in Standardbred Racehorses

**DOI:** 10.3390/ani12223228

**Published:** 2022-11-21

**Authors:** Chiara Maria Lo Feudo, Luca Stucchi, Bianca Conturba, Giovanni Stancari, Francesco Ferrucci

**Affiliations:** 1 Equine Sports Medicine Laboratory “Franco Tradati”, Department of Veterinary Medicine and Animal Sciences, University of Milan, 26900 Lodi, Italy; 2 Equine Unit, Veterinary Teaching Hospital, Department of Veterinary Medicine and Animal Sciences, University of Milan, 26900 Lodi, Italy

**Keywords:** equine asthma, equine respiratory medicine, equine sports medicine, equine performance, treadmill test, poor performance, aerobic capacity

## Abstract

**Simple Summary:**

Among racehorses, mild–moderate equine asthma (MEA) represents the second most frequent cause of poor performance. Multiple authors have tried to identify the direct effects of tracheal mucus accumulation and the cytological profile of the bronchoalveolar lavage (BAL) on athletic capacity, obtaining contrasting results. The present retrospective study aims to investigate the associations between different signs of airway inflammation and a series of fitness parameters obtained through a standardized incremental treadmill test in a population of 116 poorly performing Standardbred racehorses. The possible relationships between treadmill parameters and endoscopic scores, BAL leukocyte populations, and bacterial cultures of tracheal wash were statistically evaluated. The percentage of neutrophils in the BAL was correlated with several fitness parameters, negatively affecting aerobic capacity; analogously, horses with neutrophilic or mixed MEA showed worse athletic capacity compared with those with eosinophilic–mastocytic MEA. These results suggest that lower airway neutrophilia negatively affects sports performance.

**Abstract:**

Mild–moderate equine asthma (MEA) is a common respiratory disorder among racehorses, characterized by lower airway inflammation. Although it is generally agreed that MEA causes poor performance, contrasting results have been reported about the effects of tracheal mucus and the leukocyte populations of the bronchoalveolar lavage (BAL) on performance. This study aims to investigate the associations between airway inflammation and fitness parameters measured during an incremental treadmill test on Standardbred racehorses. For this purpose, the clinical records of 116 Standardbreds subjected to a diagnostic protocol for poor performance were retrospectively reviewed. Parametric and nonparametric statistics were used to evaluate the relationships between endoscopic scores, BAL cytological results, and fitness parameters. Moreover, horses were divided into MEA and non-MEA groups and into neutrophilic, eosinophilic–mastocytic, and mixed MEA groups; fitness parameters were compared between groups. Neutrophils percentages were inversely correlated with the speed at a heart rate of 200 bpm, the speed and heart rate at a plasma lactate concentration of 4 mmol/L (VLa4 and HRLa4), and the maximal speed, while they were positively correlated with higher lactate concentrations. Moreover, significant differences were detected between different MEA subtypes concerning VLa4, HRLa4, and lactate concentrations. These results suggest that airway neutrophilic inflammation impairs athletic capacity in Standardbreds.

## 1. Introduction

The racehorse industry is spread worldwide, with important economic, social, and employment implications in many countries. During the last few years, the attention to horse welfare and health protection has increased [1]; therefore, research on disorders typically affecting racehorse health and performance is of special interest. There is evidence that the respiratory system represents the main performance-limiting factor in racehorses undergoing maximal and supramaximal exercise [2]; in particular, oxygen delivery during strenuous exercise primarily limits the increase in maximal oxygen consumption (VO_2_ max) [3], which reflects the aerobic capacity and, therefore, the athletic potential of the horse [4]. As a consequence, any disorder of the respiratory system may affect aerobic metabolism, finally leading to sports performance impairment [3].

Among young racehorses, mild–moderate equine asthma (MEA) is a common inflammatory disease of the lower airway, characterized by mucus hypersecretion, airflow obstruction, and airway hyperreactivity. Affected horses commonly show chronic, occasional cough, serous to mucopurulent nasal discharge, and poor performance [5]. However, the effects of MEA on performance can be difficult to define and quantify. Previous studies rely either on trainers’ or owners’ expectations and impressions [6,7,8], race placements [9,10,11,12], or fitness parameters measured during standardized exercise tests [13,14,15,16,17,18]. Probably due to the different study designs, the literature includes contrasting results about the specific effects of lower airway inflammation—either identified as tracheal mucus accumulation or increased inflammatory cells in tracheal wash (TW) or bronchoalveolar lavage (BAL) samples—on the athletic capacity of horses.

In some studies, a high degree of mucus accumulation in the trachea has been associated with lower race positions in both Thoroughbred [9] and Standardbred racehorses [19] and with performances below expectations in Standardbreds [16]; conversely, other studies have found no association between tracheal mucus score and racing results in Thoroughbreds [11,12].

Neutrophilia of the BAL has been associated with exercise intolerance [8] and unsatisfactory racing results in Thoroughbreds [10] and Standardbreds [16]; moreover, one study reported a strong negative association between BAL mastocytosis and racing placements [10]. Some other studies investigated the relationship between BAL cytological profile and fitness parameters measured during incremental exercise tests on a treadmill; while BAL neutrophilia was associated with a lower aerobic–anaerobic threshold in one study on Standardbreds [18], no relationship between BAL differential cell count and several fitness parameters was observed by other authors in Standardbred or in Thoroughbred racehorses [14,15]. Instead, when the authors considered the cytological results of the TW, associations with racing results were lacking in Thoroughbreds [9,11], while one study reported a negative impact from TW neutrophilia on performance in Standardbreds [16].

Overall, to date, no consistent evidence for relationships between performance and tracheal mucus accumulation and between performance and BAL cytological profile has been found in racehorses [20]. The present study aims to investigate the impact of lower airway inflammation on athletic capacity in Standardbred racehorses by evaluating the associations of the findings of both airway endoscopy and BAL cytology with a wide range of fitness parameters obtained during a standardized incremental testing protocol on a treadmill.

## 2. Materials and Methods

### 2.1. Study Population

For the present study, we retrospectively reviewed the clinical records of Standardbred racehorses referred to the Equine Unit of the Veterinary Teaching Hospital of the University of Milan (Italy) for poor performance evaluation over a period of 20 years, from 2002 to 2021.

Ethical review and approval were waived since only clinical patients were included and underwent all procedures exclusively for diagnostic purposes; moreover, informed consent for the use of clinical data was obtained from all owners or holders.

All included horses (*n* = 116) were in full training upon admission and were subjected to a complete diagnostic protocol, which included collection of history, clinical examination and lameness evaluation, laboratory analyses, electrocardiogram at rest, upper airway endoscopy at rest, incremental treadmill exercise test, high-speed treadmill endoscopy, tracheobronchoscopy 30 min post-exercise for the evaluation of exercise-induced pulmonary hemorrhage, TW and BAL collection with a cytological examination of the BAL, and a microbiological examination of the TW [21].

Horses affected by other diseases that could affect athletic performance, including systemic illness, lameness, clinically significant cardiac arrhythmias or valvular regurgitation, or dynamic upper airway obstructions or rhabdomyolysis, were excluded from the study.

### 2.2. Incremental Metabolic Test on Treadmill

After one day of being acclimated to a high-speed treadmill (Sato I, Uppsala, Sweden), after one or two training sessions, the horses underwent an incremental treadmill test. An inclination of 5% was applied to the belt: after a warmup phase (4 min walking at 1.5 m/s and 3 min trotting at 6 m/s), the protocol consisted of 1 min phases, increasing the speed by 1 m/s until the onset of fatigue. At the end of the test, horses were cooled down by a 30 min walk with 0% slope. Throughout the duration of the test, horses were wearing a heart rate monitor (Polar, Equine Inzone FT1, Steinhausen, Switzerland), and a continuous ECG was obtained before, during, and after exercise using a Holter recorder (Custo Flash 200, Sylco, Monza, Italy; Cardioline^®^ Click Holter, Trento, Italy). Blood samples were collected through an extension tube connected to a 14 G Teflon catheter placed in the left jugular vein at different timepoints: at rest, at the end of the warmup phase and of each speed phase, and 1, 5, 15, and 30 min postexercise. Blood samples of 1 mL each were transferred into tubes containing 10 mg sodium fluoride and 2 mg potassium oxalate, centrifugated within 15 min, and refrigerated. Plasma lactate values were obtained using the enzymatic colorimetric method with a lactate dry-fast kit due to the automatic system and its proper reagents (Uni Fast System II Analyzer, Sclavo, Italy). In some horses, at each phase, an aliquot of blood was transferred into heparinized syringes, and blood pH (*n* = 69) and hematocrit (*n* = 46) were measured using a blood gas analyzer (IL 1630, Instrumentation Laboratory, Milan, Italy; Opti CCA, Opti Medical System, Roswell, USA) [22].

The fitness parameters obtained during the incremental treadmill test included:

- V200: Speed at a heart rate of 200 bpm;
- VLa4: Speed at a plasma lactate concentration of 4 mmol/L;
- HRLa4: Heart rate at a plasma lactate concentration of 4 mmol/L;
- Lac_max_: Maximum plasma concentration of lactate reached during the test or cool down;
- V_max_: Maximum speed reached during the test (until the horse became fatigued);
- Lac_1, 5, 15, 30_: Plasma lactate concentrations at 1, 5, 15, and 30 min postexercise, during the cool down;
- HR_1, 5, 15, 30_: Heart rate at 1, 5, 15, and 30 min post-exercise, during the cool down;
- pH_min_: Minimum pH measured during or after the test;
- Ht_max_: Maximum hematocrit measured during or after the test.

A specific software (Lactate-E 1.0, Dr. David Higgins), which provides precise lactate threshold markers via inverse prediction [23], was used to calculate exact values of VLa4 and HRLa4.

### 2.3. Airway Endoscopy and Collection of TW and BAL

After one day of active rest (hand walking), horses underwent high-speed treadmill endoscopy and postexercise tracheobronchoscopy. At least twenty-four hours later, airway endoscopy with TW and BAL collection was performed. To this end, horses were contained in a stock and sedated with detomidine hydrochloride (0.01 mg/kg IV). A flexible videoendoscope (ETM PVG-325, Storz, Tuttlingen, Germany; EC-530WL-P, Fujifilm, Tokyo, Japan) was passed through the left nasal passage, and the upper and lower tracts of the respiratory system were visualized [24].

Endoscopic scores were assigned to

- Pharyngeal lymphoid hyperplasia (PLH), from 0 to 4 [25];
- Tracheal mucus accumulation (TM), from 0 to 5 [26];
- Tracheal bifurcation blunting (TB), from 0 to 4 [27].

A TW was performed by flushing a 60 mL prewarmed sterile saline (0.9%) into the intrathoracic portion of the tracheal lumen through a sterile single-lumen catheter advanced through the biopsy channel of the endoscope; at the level of the curvature of the distal trachea, the fluid deposit was re-aspirated and transferred into sterile plain tubes for microbiological evaluation. Immediately following the TW, a BAL sample was collected for cytological examination. The endoscope was advanced to the tracheal bifurcation, where 60 mL of a 0.5% lidocaine hydrochloride solution was sprayed through a sterile single-lumen catheter inserted into the biopsy channel of the endoscope; as the coughing reflex was inhibited, the endoscope was furtherly advanced into the bronchial tree. Once the endoscope was wedged within a segmental bronchus, a 300 mL sterile saline (0.9%) was instilled, and the fluid was immediately aspirated. The collected BAL was stored in sterile EDTA tubes and subjected to processing within 90 min [28].

### 2.4. Cytological Examination of the BAL and Microbiological Examination of the TW

To perform cytological examination, 300 µL of pooled BAL was cytocentrifugated (Rotofix 32, Hettich Cyto System, Tuttlingen, Germany) at 26 *g* for 5 min. The slides were air dried, stained with May–Grünwald–Giemsa and Perl’s Prussian blue, and observed under a light microscope at 400× and 1000× for a 400-cell leukocyte differential count. To perform the microbiological examination, 10 µL of TW were cultured on blood agar plates (5%) and incubated at 37 °C; after 48 h, bacterial species identification and CFU/mL count were performed [28].

### 2.5. Group Allocation

Based on the results obtained with BAL cytology, horses were divided into groups following two different models.

In the first model, horses were divided into MEA and non-MEA groups: in particular, horses were considered affected by MEA when BAL cytology consisted of neutrophils > 10% and/or eosinophils > 5% and/or mast cells > 5%. In this case, we decided to adopt these wider cutoff values because 115/116 horses showed an increase in at least one population of inflammatory cells in the BAL compared with normal values (neutrophils ≤ 5%, eosinophils ≤ 1%, mast cells ≤ 2%). The BAL cutoff values used in this model are those considered unequivocally consistent with MEA regardless of the technique used for cytological examination [5].

In the second model, horses were divided into neutrophilic MEA, eosinophilic–mastocytic MEA, and mixed MEA groups based on the following inclusion criteria [15,29]:

- Neutrophilic MEA (n-MEA): neutrophils > 5%, eosinophils ≤ 1%, mast cells ≤ 2%;
- Eosinophilic–mastocytic MEA (e-MEA): neutrophils ≤ 5%, eosinophils > 1%, and/or mast cells > 2%;
- Mixed MEA (m-MEA): neutrophils > 5%, eosinophils > 1% and/or mast cells > 2%.

In this model, stricter cutoff values were adopted in order to distinguish the different MEA subtypes. Only one horse presented all BAL cell types within normal limits and was not included in this classification.

### 2.6. Statistical Analysis

The data obtained by the present study were analyzed using descriptive statistics and evaluated for normality through the Shapiro–Wilk test. The associations between endoscopic scores (PLH, TM, and TB) and fitness parameters were evaluated with the Spearman correlation. Analogously, the relationships between BAL cell-type percentages and fitness parameters were investigated using Spearman correlation or simple linear regression according to data distribution. All fitness parameters and BAL cytology results were then compared between MEA and non-MEA groups by means of the Mann–Whitney test or the unpaired t-test and between the n-MEA, e-MEA, and m-MEA groups by means of the Kruskal–Wallis test and Dunn’s multiple comparisons test or ordinary ANOVA and Fisher’s LSD test based on data distribution. Moreover, fitness parameters were compared between horses with TW-positive and -negative bacterial cultures through the Mann–Whitney or unpaired t-tests. The associations between age and endoscopic scores, BAL cytology, and fitness parameters were evaluated by means of Spearman correlation. The possible influence of sex on fitness parameters was evaluated with the Mann–Whitney test. Finally, age and sex were compared between MEA and non-MEA groups using, respectively, the Mann–Whitney test and Fisher’s exact test and between n-MEA, e-MEA, and m-MEA by means of the Kruskal–Wallis test, Dunn’s multiple comparisons test, and the chi-square test.

Normally distributed data are displayed as mean ± standard deviation (SD), while non-normally distributed data are expressed as median and interquartile range (IQR). Statistical significance was set at *p* < 0.05. Data were analyzed using a commercially available statistical software package (GraphPad Prism 9.4.1 for MacOS; GraphPad Software, San Diego, CA, USA).

## 3. Results

### 3.1. Horses

Among the Standardbred racehorses that were subjected to the diagnostic protocol described above, between 2002 and 2021, 116 patients met the inclusion criteria of the present study. The study population included 76 males (63 stallions and 13 geldings) and 40 females, ranging in age from 2 to 8 years (median 3, IQR 3–4 years). The MEA group included 80 horses (10 geldings, 46 stallions, 24 mares), from 2 to 8 years old (median 3, IQR 3–5 years), while the non-MEA group included 36 horses (3 geldings, 17 stallions, 16 mares), from 2 to 6 years old (median 3, IQR 3–4 years). The two groups were age- and sex-matched. The n-MEA group included 14 horses (2 geldings, 6 stallions, 6 mares), ranging in age from 2 to 8 years (median 3, IQR 3–4 years); the e-MEA group included 36 horses (3 geldings, 25 stallions, 8 mares), from 2 to 7 years old (median 3.5, IQR 3–4.75); the m-MEA group included 66 horses (8 geldings, 32 stallions, 26 mares), from 2 to 7 years old (median 3, IQR 3–4). The three groups were age- and sex-matched.

### 3.2. Endoscopic Scores, BAL Cytology, and TW Microbiology

Among the study population, a median PLH score of 2 (IQR 1–2), a median TM score of 1 (IQR 1–2), and a median TB score of 1 (IQR 1–2) were observed.

The results of the BAL cytological examination in the study population and in different groups are shown in Table 1. Lymphocyte percentages were lower (*p* = 0.0001) and neutrophil percentages were higher (*p* < 0.0001) in the MEA group compared with the non-MEA group. Lymphocyte percentages were higher and neutrophil percentages were lower in the e-MEA group compared with the n-MEA (lymphocytes, *p* = 0.0002; neutrophils, *p* < 0.0001) and the m-MEA (lymphocytes, *p* < 0.0001; neutrophils, *p* < 0.0001) groups. Eosinophil and mast cell counts were lower in the n-MEA group compared with the e-MEA (eosinophils, *p* = 0.0008; mast cells, *p* < 0.0001) and the m-MEA (eosinophils, *p* = 0.0098; mast cells, *p* < 0.0001) groups.

The bacterial culture of TW was negative in 62 horses (53.45%) and positive in the remaining 54 horses (46.55%); the most commonly isolated bacteria were *Streptococcus* spp. (39 horses, 72.22%), *Klebsiella* spp. (6 horses, 11.11%), and *Pasteurella* spp. (5 horses, 9.26%).

### 3.3. Fitness Parameters

The results regarding the fitness parameters measured during the incremental treadmill test in the study population; the MEA and non-MEA groups; and the n-MEA, e-MEA, and m-MEA groups are displayed in Table 2.

### 3.4. Statistical Results

The PLH score was inversely correlated with HR_5_ (*p* = 0.0322, r = −0.21), HR_15_ (*p* = 0.0214, r = −0.22), HR_30_ (*p* = 0.0218, r = −0.22), and Ht_max_ (*p* = 0.0009, r = −0.47). The TM score was inversely correlated with Ht_max_ (*p* = 0.021, r = −0.34). No association was observed between the TB score and any fitness parameter.

The percentage of macrophages in the BAL was inversely correlated with VLa4 (*p* = 0.0259, r = −0.21), HRLa4 (*p* = 0.0014, r = −0.29), HR_15_ (*p* = 0.0329, r = −0.21), and HR_30_ (*p* = 0.0441, r = −0.19).

The lymphocyte count was positively correlated with VLa4 (*p* = 0.0003, r = 0.33), HRLa4 (*p* = 0.0008, r = 0.31), and HR_15_ (*p* = 0.0379, r = 0.20), while inversely correlated with Lac_max_ (*p* = 0.0290, r = −0.20), Lac_1_ (*p* = 0.0231, r = −0.22), Lac_5_ (*p* = 0.0146, r = −0.24), Lac_15_ (*p* = 0.0142, r = −0.24), and Lac_30_ (*p* = 0.0024, r = −0.29).

The percentage of neutrophils was inversely correlated with V200 (*p* = 0.0466, r = −0.19), VLa4 (*p* < 0.0001, r = −0.35), HRLa4 (*p* = 0.0083, r = −0.24), and V_max_ (*p* = 0.0241, r = −0.21) and positively correlated with Lac_max_ (*p* = 0.0011, r = 0.30), Lac_1_ (*p* = 0.0017, r = 0.30), Lac_5_ (*p* = 0.0013, r = 0.31), Lac_15_ (*p* = 0.0032, r = 0.28), and Lac_30_ (*p* = 0.0007, r = 0.32).

No association was observed between eosinophil and mast cell percentages and any fitness parameter. Similarly, the microbiological results of the TW were not related to any fitness parameter.

When dividing the population into the MEA and non-MEA groups, no differences were observed between groups for any fitness parameters. In contrast, when dividing the population into n-MEA, e-MEA, and m-MEA groups, several differences in fitness parameters were detected between groups. In particular, VLa4 was higher in the e-MEA group compared with the n-MEA (*p* = 0.0112) and the m-MEA (*p* = 0.0050) groups; the HRLa4 was also higher in the e-MEA group compared with the m-MEA group (*p* = 0.0103). The values of Lac_max_ and Lac_1_ were lower in the e-MEA group compared with the n-MEA (Lac_max_, *p* = 0.0223; Lac_1_, *p* = 0.0333) and the m-MEA (Lac_max_, *p* = 0.0179; Lac_1_, *p* = 0.0249) groups; finally, Lac_5_ and Lac_15_ were statistically lower in the e-MEA group compared with the m-MEA group (Lac_5_, *p* = 0.0356; Lac_15_, *p* = 0.0180).

Age showed a positive correlation with BAL mast cells percentages (*p* = 0.0342, r = 0.20), VLa4 (*p* = 0.0197, r = 0.22), V_max_ (*p* = 0.0001, r = 0.35), and Ht_max_ (*p* = 0.0034, r = 0.42); conversely, an inverse correlation was observed between age and BAL eosinophil counts (*p* = 0.0287, r = −0.20), PLH score (*p* < 0.0001, r = −0.50) and the TM score (*p* = 0.0239, r = −0.21). Compared with females, males had higher V200 (*p* = 0.0081), VLa4 (*p* = 0.0047), HRLa4 (*p* = 0.0137), and V_max_ (*p* = 0.0382) and lower Lac_max_ (*p* = 0.0204), Lac_1_ (*p* = 0.0132), and Lac_5_ (*p* = 0.0123). No differences in age or sex distribution were detected between the MEA and non-MEA groups, nor between the n-MEA, e-MEA, and m-MEA groups.

## 4. Discussion

Mild–moderate equine asthma is considered one of the most common causes of poor performance in young racehorses, second only to orthopedic disorders [30,31]. Several studies have attempted to identify and measure the effects of lower airway inflammation on the athletic capacity and consequent sports performance of racehorses. However, to date, the evidence for a relationship between MEA and performance is not yet consistent [19]. The present study aimed to evaluate associations between the findings of airway endoscopy, BAL cytology, and TW bacteriology with a series of fitness parameters measured during a standardized incremental treadmill test in a population of poorly performing Standardbred racehorses. Briefly, only neutrophilic inflammation in the lower airway proved to negatively affect aerobic capacity, while tracheal mucus, BAL mastocytosis and eosinophilia, and bacterial tracheal colonization did not seem to be associated with decreased athletic performance.

In previous studies, the associations between lower airway inflammation and performance were investigated either by evaluating BAL or TW cytology and microbiology and airway endoscopic scores or by dividing horses into MEA-affected and not-MEA-affected groups; performance assessment relied either on trainers’ or owners’ impressions, racing results, or fitness parameters measured during standardized exercise testing. In the present study, we decided to take into consideration a complete assessment of airway inflammation, including the endoscopic scores of PLH, TM, and TB; a cytological examination of the BAL; and a microbiological examination of the TW.

Concerning endoscopic scores, in our study, horses with higher PLH scores showed lower heart rates during the recovery period; the clinical meaning of this association is unsure, as it may be the consequence of the less-demanding physical work undergone by young horses. In fact, it is known that PLH is more severe in younger horses and tends to decrease naturally with age [9,28,32,33,34]; accordingly, in our study, younger horses had higher PLH scores and reached a lower maximal speed, and this could explain why horses with higher PLH scores recovered faster. Few studies had previously investigated the possible relationship between the severity of PLH and sports performance, and none of them reported any association in racehorses or in dressage and show-jumping horses [6,9,12,16,33,34], suggesting that PLH does not impact athletic capacity; only in one study on endurance horses was more severe PLH observed in poorly performing horses compared with controls [35]. In the present study, higher scores for both PLH and TM were associated with a lower maximum hematocrit. It is known that, during exercise, the oxygen-carrying capacity of the horse’s blood increases proportionally with the rise in hematocrit; however, postexercise hematocrit failed to be associated with performance in a previous study [36]. Therefore, the clinical meaning of this finding and its effects on performance are unclear, as the lower Ht observed in our study may be related to the younger age of horses showing higher PLH and TM scores. Interestingly, in our study, the TM score was not associated with any other fitness parameter, suggesting that no direct effect of mucus accumulation on decreased athletic capacity exists; nevertheless, the median and interquartile ranges of the TM score were low, indicating that most horses in the study population only showed a little or moderate accumulation of tracheal mucus, while a more marked accumulation was rarely observed. Therefore, the paucity of horses with high TM scores may have prevented us from highlighting any significant correlation between tracheal mucus accumulation and fitness parameters; as a matter of fact, some previous studies reported an impact from mucus accumulation on performance only when present in large amounts [6,9]. In general, an association between TM scores and poor performance was reported by numerous studies on Standardbred trotters [16,19], Thoroughbred racehorses [9], endurance horses [35], and dressage and show-jumping horses [6]. However, in two studies on Thoroughbreds, no association between TM score and racing results was detected [11,12], in accordance with our findings. Therefore, consistent evidence about the impact of TM accumulation on performance is still lacking [20]. In the present study, the blunting of the tracheal bifurcation was also considered, but no association between the TB score and any fitness parameters was found; to the best of the authors’ knowledge, no studies have previously investigated its possible association with sports performance. In the case of TB, scores were generally low among our population, probably because thickening and edema of the tracheal bifurcation are more typical of severe equine asthma [28,32,37,38].

When considering the cytology of the BAL, it is known that neutrophilia is a common finding among poorly performing racehorses [31]. In the present study, we found significant effects caused by the percentage of neutrophils on several fitness parameters: in particular, as neutrophils increased, the values of V200 and VLa4 and the maximal exercise speed decreased, while the peak of plasma lactate concentration and plasma lactate concentrations during recovery time increased. These results suggest that neutrophilic inflammation in the lower airway impairs aerobic capacity, probably by interfering with gas exchanges at the alveolar–capillary level; as a consequence, hypoxemia may occur at lower speeds, with an earlier switch to anaerobic metabolism and prolonged lactate accumulation. Indeed, horses become fatigued at lower speeds, and high lactate concentrations remain longer after the end of the exercise. A number of previous studies reported a relationship between BAL neutrophilia and decreased performance: in particular, it was associated with exercise intolerance during strenuous exercise [8] and worse racing results in Thoroughbred racehorses [19] and with performance below expectations in Standardbred trotters [16] and endurance horses [35]. To the best of the authors’ knowledge, no studies have denied the association between BAL neutrophilia and performance when defined on the basis of trainers’ impressions or racing results. Conversely, contrasting results have been reported when investigating the effects of BAL neutrophilia on fitness parameters measured during exercise tests on treadmills. In a study on Standardbreds, the cytological findings of the BAL were not associated with any indices of athletic capacity, including V200, VO_2_ max, maximal carbon dioxide production, respiratory quotient, and the number of steps completed during the treadmill test [14]. Similarly, another study including Thoroughbred and Standardbred racehorses reported no relationship between BAL cytology and blood gas values, the peak of lactate concentration, and minimum pH measured during treadmill exercise [15]. Only a preliminary study by our research group observed an association between BAL neutrophilia and a lower aerobic–anaerobic threshold in Standardbreds [18]. Therefore, the present study is the first one that highlights any measurable effects of BAL neutrophilia on physiological responses to treadmill exercise in horses. It is worth mentioning that macrophage and lymphocyte percentages were also correlated with some fitness parameters; however, it is hard to hypothesize a causative relationship, as the relative percentages of these cell populations may increase or decrease as a consequence of neutrophil variations. Finally, no association between eosinophil and mast cell percentages and any fitness parameters was observed; while no studies have reported a relationship between BAL eosinophils and performance, BAL mastocytosis has been shown to be strongly correlated with worse racing results and a decreased likelihood of winning in a study on Thoroughbred racehorses, impairing performance even to a greater degree compared with BAL neutrophilia [10]. No other studies have reported a similar association, which certainly deserves further investigation. Interestingly, some previous studies investigated the relationship between performance and the cytological profile of the TW. Two studies, based on racing results, detected no association in Thoroughbred racehorses [9,11]; in particular, in one of them, horses that actually raced showed higher TW neutrophil percentages compared with those that did not race, suggesting that the increase in tracheal neutrophils may be a consequence of intense exercise [9,13,39]. Probably for the same reason, in dressage and show-jumping horses, tracheal neutrophilia was associated with a strong will to perform and a better general impression [6]. Conversely, other authors reported a relationship between higher percentages of neutrophils in the TW and poor performance in Standardbreds [16] and endurance horses [35]. The discordance between these studies may be due to the fact that tracheal neutrophilia does not necessarily reflect neutrophilic inflammation within the lung, as the lack of correlation between TW and BAL cytology has been widely documented [40,41]; in fact, for this reason, the cytology of the TW is considered inappropriate for the diagnosis of MEA, which should rely on BAL cytological examination [5].

Besides the evaluation of the direct effects of BAL inflammatory cells on fitness parameters, we also decided to compare them between horses affected and not affected by MEA and between different MEA subtypes. Previous studies, which classified horses merely based on the diagnosis of MEA, reported variable results about its effects on fitness parameters. In particular, two studies reported a more pronounced exercise-induced hypoxemia in MEA horses [13,17], while other authors observed no difference in PaO_2_ during a treadmill test between healthy horses and horses with lower airway inflammation [42]. Regarding lactate concentration, MEA was not associated with VLa4 [13,17] or with lactate concentrations at different steps of the treadmill test [17,42] in previous studies; conversely, MEA horses reached higher peaks of lactate in one study [13] but not in another one [17]. In a study reporting an association with maximum lactate, lactate concentrations during recovery time were also higher in MEA horses, while blood pH and bicarbonate concentrations were lower; moreover, no associations with V200 and the maximal speed were observed [13]. In our study, no differences between MEA and non-MEA horses were detected for any fitness parameter; it must be underlined that we selected the suggested wide cutoff values [5]; this could have prevented mildly asthmatic horses from being recognized as MEA-affected. The possible allocation of these “borderline” subjects in the healthy group may not have allowed us to detect significant differences between MEA and non-MEA groups. Unfortunately, we could not have used stricter cutoff values, as almost all the horses in our population would have been allocated in the MEA group. In general, MEA affects up to 80% of racehorses in training [10,43], but its higher prevalence in our population was probably biased by the exclusive inclusion of poorly performing horses. Therefore, in our population, no healthy control horses could have been included, which represents the main limitation of the present study. Nevertheless, to overcome this limit, we divided the population based on MEA subtypes: neutrophilic, eosinophilic–mastocytic, and mixed [5]; indeed, different cytological profiles of MEA may reflect different etiopathogenetic mechanisms and implications for performance, as previously hypothesized by various authors [10,15]. Our study revealed multiple differences in fitness parameters between horses with eosinophilic–mastocytic MEA and horses with either neutrophilic or mixed MEA, confirming this hypothesis; moreover, this finding once again proved that neutrophilic inflammation was the main cause of decreased athletic capacity in our study population.

As the role of bacteria in MEA has long been discussed [44,45,46,47], we also examined the possible relationship between the results of TW bacterial cultures and fitness parameters; however, we did not find any significant association. Only one previous study reported a negative impact of bacterial colonization on performance in racehorses, but in that study, the microbiological examination was performed on the BAL [8]. In our study, bacteria were isolated from the TW: bacterial colonization in the trachea does not necessarily implicate lung neutrophilia [28], and low bacterial counts could even be the result of sample contamination during endoscopy, which is not uncommon in MEA-affected horses [45]. Moreover, the isolation of bacteria is frequent in young racehorses due to the immaturity of their immunity [28,45]. Therefore, results of microbiological examinations should be interpreted cautiously when considering a possible relationship with performance.

Finally, the effect of age and sex on fitness parameters was also noticed. In particular, age was positively correlated with VLa4 and the maximal speed reached during the treadmill test; an explanation for these results may be that adult horses are probably fitter and more physically mature, in agreement with previous studies [21,48]. Similarly, males showed better fitness parameters compared with females, in agreement with previous reports [21,49,50]. However, as all groups were age and sex-matched, it is likely that the contribution of age and sex to influencing fitness parameters was equal among different groups and that detected differences between MEA subtypes are exclusively attributable to lower airway inflammation.

The results of the present study open the door to the possibility of further investigating the possible relationship between airway inflammation and other innovative parameters related to exercise response. In fact, over the last few years, some novel biomarkers have been identified for monitoring physiological responses to training in horses. In particular, multiple studies have reported an increase in circulating levels of acute phase proteins, such as serum amyloid A, associated with exercise intensity and the level of fitness of the horse, which may be useful for the detection of subclinical pathologies affecting performance [51,52]. Moreover, oxidant and antioxidant parameter assessment may aid in the evaluation of fitness in racehorses [53,54]. Finally, a recent preliminary study reported a correlation between infrared thermography performed at the level of the *musculus trapezius pars thoracica* region and blood lactate values during race training, suggesting its possible applicability in future studies to obtain useful fitness measurement in a completely noninvasive way [55].

## 5. Conclusions

The present study confirms that lower airway neutrophilia negatively affects athletic performance in Standardbred racehorses by impairing aerobic capacity and causing a prolonged accumulation of lactate, probably due to inefficient gas exchanges at the alveolar–capillary level. In particular, the affected fitness parameters include a lower speed at a heart rate of 200 bpm, a lower speed and heart rate at the aerobic–anaerobic threshold, higher peak lactate, and prolonged lactate accumulation during recovery. Conversely, eosinophilic and mastocytic lung inflammation does not seem to interfere with any fitness parameters, nor does the possible concomitant bacterial colonization in the trachea. Endoscopic signs of upper and lower airway inflammation, including pharyngeal lymphoid hyperplasia, tracheal mucus accumulation, and edema of the tracheal bifurcation, do not represent risk factors for decreased athletic capacity. In conclusion, the results of the present study suggest that the association between mild–moderate equine asthma and poor performance in racehorses should be attributed to neutrophilic inflammation, while the role of other signs of airway inflammation should be interpreted cautiously and probably need further investigation.

## Figures and Tables

**Table 1:** Results of the cytological examination of the BAL in the whole study population; the MEA and the non-MEA groups; and the n-MEA, e-MEA, and m-MEA groups. Data are expressed as mean ± standard deviation if normally distributed and as median (interquartile range) if not normally distributed. Data on lines with different superscript letters are significantly different from each other (*p* < 0.05).

Cell Type (%)	Population	MEA	Non-MEA	n-MEA	e-MEA	m-MEA
Macrophages	44.79 ± 9.33	43.79 ± 9.25	47.03 ± 9.23	46.43 ± 6.35	41.86 ± 9.52	46.05 ± 9.49
Lymphocytes	36.1 ± 12.25	33.25 ± 12.14 ^a^	42.44 ± 10.05 ^b^	32.36 ± 10.29 ^a^	45.19 ± 8.71 ^b^	31.94 ± 11.71 ^a^
Neutrophils	10 (5–17)	14 (7–20) ^a^	5 (3.25–8) ^b^	18.5 (13.75–30.25) ^a^	3 (3–4.75) ^b^	13.5 (8.75–20) ^a^
Eosinophils	1 (0–3)	1 (0–4.75)	1 (0–2)	0 (0–1) ^a^	2 (0–5.75) ^b^	1 (0–3) ^b^
Mast cells	4 (3–6)	5 (3–7)	4 (3–5)	2 (1–2) ^a^	5 (4–6.75) ^b^	4 (3–6) ^b^

**Table 2:** Fitness parameters obtained through the incremental test on a treadmill in the whole study population; the MEA and the non-MEA groups; and the n-MEA, e-MEA, and m-MEA groups. Data are expressed as mean ± standard deviation if normally distributed and as median (interquartile range) if not normally distributed. Data on lines with different superscript letters are significantly different from each other (*p* < 0.05).

Variable	Population	MEA	Non-MEA	n-MEA	e-MEA	m-MEA
V200 (m/s)	8 (7–8.5)	8 (7–9)	8 (7–8.38)	7 (6.38–8.25)	8 (7.5–8.5)	8 (6.37–8.63)
VLa4 (m/s)	8.45 (7.15–9.3)	8.4 (7.03–9.35)	8.75 (7.65–9.3)	8.05 (6.63–8.75) ^a^	9.1 (8.03–9.7) ^b^	8.2 (7–9.13) ^a^
HRLa4 (bpm)	206 (198–214)	204.8 (197.6–211.2)	210.2 (198.9–216.2)	199.1 (193.8–215.8)	210.6 (202.1–216.7) ^a^	204.1 (195.7–210.9) ^b^
Lac max (mmol/L)	20.71 ± 6.89	21.36 ± 6.99	19.27 ± 6.49	23.12 ± 4.89 ^a^	18.22 ± 5.79 ^b^	21.56 ± 7.46 ^a^
Lac 1 (mmol/L)	19.26 ± 6.07	19.62 ± 6.09	18.41 ± 6.03	21.42 ± 5.69 ^a^	17.13 ± 5.07 ^b^	20.01 ± 6.39 ^a^
Lac 5 (mmol/L)	20.14 ± 6.94	20.77 ± 6.99	18.65 ± 6.69	21.79 ± 4.99	17.93 ± 6.08 ^a^	21.04 ± 7.47 ^b^
Lac 15 (mmol/L)	16.8 ± 7.85	17.58 ± 8.02	14.95 ± 7.22	17.94 ± 4.27	14.11 ± 6.62 ^a^	18.06 ± 8.68 ^b^
Lac 30 (mmol/L)	10.77 (6.36–15.27)	11.71 (7.54–15.97)	9.07 (4.9–14.49)	11.24 (8.73–14.39)	8.38 (4.29–13.82) ^a^	11.83 (7.76–15.83) ^b^
HR 1 (bpm)	152 ± 18.64	150.7 ± 19.19	154.9 ± 17.2	153.8 ± 18.55	150 ± 18.8	152.7 ± 18.78
HR 5 (bpm)	117.5 (107.3–127.8)	117 (108.3–127.5)	119 (106–127.8)	122 (111.5–130)	120 (110.8–128.5)	117 (105.8–125.3)
HR 15 (bpm)	93.41 ± 17.1	92.74 ± 15.64	95 ± 20.32	94.75 ± 12.9	98.5 ± 18.43 ^a^	90.35 ± 16.57 ^b^
HR 30 (bpm)	74.8 ± 18.14	75.74 ± 17.68	72.56 ± 19.31	78.75 ± 14.54	74.71 ± 19.34	74.09 ± 18.26
V max (m/s)	11 (11–11)	11 (11–12)	11 (11–11)	11 (10.75–11)	11 (11–11.75)	11 (11–12)
Ht max (%)	64.78 ± 4.03	64.86 ± 3.89	64.44 ± 4.8	65.11 ± 3.52	64.80 ± 3.11	64.69 ± 4.37
pH min	7.16 ± 0.1	7.16 ± 0.1	7.15 ± 0.1	7.15 ± 0.1	7.14 ± 0.08	7.16 ± 0.11

## Data Availability

The data presented in this study are available upon request from the corresponding author.
